# Effects of Job Content and Physical Activity on Body Mass Index among Obese Managers of the Mexican Manufacturing Industry

**DOI:** 10.3390/ijerph17113969

**Published:** 2020-06-03

**Authors:** Oziely Daniela Armenta-Hernandez, Aidé Aracely Maldonado-Macias, Margarita Ortiz Solís, Miguel Ángel Serrano-Rosa, Yolanda Angélica Baez-López, Juan Luis Hernández-Arellano

**Affiliations:** 1 Departamento de Eléctrica y Computación, Universidad Autónoma de Ciudad Juárez, Ciudad Juárez 32310, Chihuahua, Mexico; al164439@alumnos.uacj.mx; 2 Departamento de Ingeniería Industrial y Manufactura, Universidad Autónoma de Ciudad Juárez, Ave. del Charro 450 Norte, Ciudad Juárez 32310, Chihuahua, Mexico; al164612@alumnos.uacj.mx; 3 Departamento de Psicobiología, Facultad de Psicología, Universidad de Valencia, 46010 Valencia, Spain; m.angel.serrano@uv.es; 4 Facultad de Ingeniería, Arquitectura y Diseño, Universidad Autónoma de Baja California; Carretera Transpeninsular Ensenada-Tijuana 3917, Colonia Playitas, Ensenada 22860, Baja California, Mexico; yolanda@uabc.edu.mx; 5 Departamento de Diseño, Universidad Autónoma de Ciudad Juárez, Ciudad Juárez 32310, Chihuahua, Mexico; luis.hernandez@uacj.mx

**Keywords:** body mass index, job strain, obesity, physical activity

## Abstract

Mental health disorders resulting from work stressors are increasing in the Mexican manufacturing industry and worldwide. Managerial positions in these contexts are highly stressful, and although physical activity may reduce the negative effects of work stress, the relationships between these two aspects regarding their effects on the body mass index (BMI) of obese managers are scarcely studied. This article aims to study such relationships by using the Job Content Questionnaire (JCQ) dimensions with the Baecke’s physical activity questionnaire dimensions and analyzing their effects on the BMI. A sample of 255 managers from the Mexican industry, with a (BMI > 30) participated by answering the surveys and providing their weight, their height, and certain sociodemographic information. The research hypotheses were tested using WarpPLS^®^ 6.0 for structural equation modeling. The results for three models featuring acceptable reliability to estimate the direct, indirect, and total effects are presented. The first model showed a medium explanatory power, the variable of job decision-making authority having the greatest direct effect on BMI. The second model showed a medium explanatory power, and the variable of physical activity during leisure-time observed the unique direct effect on BMI. Finally, although the integrating model showed a small explanatory power, both work stress and the physical activity exerted observed direct effects on BMI reduction.

## 1. Introduction

According to the WHO [1], stress as a psychosocial work factor has both psychological and physical effects. Among the psychological effects can be anxiety, lack of concentration, and difficulty in making decisions, tiredness, and depression and sleep problems. Conversely, prestigious authors recognized that physical effects include heart diseases, digestive disorders, augmented blood pressure, headaches, and/or musculoskeletal disorders [2,3,4,5,6,7,8,9,10,11]. Work stress has become a public health problem due to new forms of work organization, new relationships and new employment patterns [12]. Theoretically, work stress requests are based on the way that human beings adapt to the demands of work [13]; other authors mention that work stress is the organism’s response to external demands [14]. In recent years, its definition is attached to the studies made by Karasek who establishes that work stress is present when the demands of work overcome humans’ control capacity [8,14]. Lately, this disorder has increased due to the constant changes generated by the creation of new companies and tasks, all of which affect workers’ health in various ways.

In addition to work stress, obesity is another important public health problem in the industrial sector due to its magnitude, rapid growth, and negative effect on the health of the population suffering from it. Obesity is known to be a systemic, chronic and multicausal disease found not only in economically developed countries [15]. The industrial sector faces significant challenges in dealing with obesity despite the various alternatives to prevent and counteract this disorder. Among other measures, Klein proposes a change of lifestyle, an increase in physical activity, and the integration of healthy eating habits into the daily routine [16].

Indeed, the variables studied in this work may be having combined effects while acting together and may also be related to each other. In recent years, illnesses caused by psychosocial factors have been more frequent and could be having an effect on employees’ wellbeing. On the other hand, fatigue, chronic stress, and harassment at work, among others, could result in an increase in absenteeism in the industrial sector [17]. Therefore, it is important to observe and study the harmful effects of work stress and obesity as they have increased cardiovascular, respiratory, and gastrointestinal problems, thus affecting both the physical and mental health of employees. In addition, the work stress and obesity-related mortality index in industrial employees has been increasing recently [18]. The reason is that the effects of work stress and obesity can also increase the risk of diabetes and some types of cancer [15] as well as of cardiovascular diseases, which are the main cause of death worldwide [19].

Among the issues mentioned, engaging in physical activity has been identified as one of the variables that most benefits the decrease and management of both stress and obesity. The Mexican Social Security Institute [20] defines physical activity as body motion that puts the muscles to work and demands energy exertion. Some authors recognize that exercise contributes to the generation of a more positive response to work stress [21]. In addition, not only does physical activity improve health, but it also helps individuals’ sense of humor and the ability to withstand difficult situations [22].

In relation to the context of this work, the industrial growth in Mexico has entailed the development of habits that lead to obesity, for example, a sedentary lifestyle, company-cooked fast food, and the lack of a fixed eating schedule. Besides, the factors that affect obesity have to do with job design. Hence, if work is deficiently designed, employees may experience an imbalance between job demands and their abilities.

Furthermore, mental health disorders related to work stressors are on the increase both in the Mexican context and abroad. Managerial job positions, specifically, are exposed to numerous work stressors, which explains their being considered among the most stressful occupations in industry [23]. Additionally, such positions are known to be highly sedentary. On the other hand, although doing physical activity may lower the effects of work stress and may contribute to reducing BMI, the relationships between physical activity, work stress and body mass index (BMI) among obese employees have received little attention [24].

Due to the above, this paper has focused on studying more extensively the relationships between these three variables as they have been deemed insufficiently addressed in the available literature. The most important contribution of this paper lies on the analysis of the relationships among work stress, physical activity and their effects on the BMI of such a vulnerable and neglected population as are the obese managers in the manufacturing industry. Additionally, the widely known Karasek’s demand–control work stress model, including the job content dimensions utilized in this research, has scarcely been related to physical activity dimensions to determine the effects of both variables (work stress and physical activity) on obese individuals. Moreover, the number of studies relating such work and physical activity dimensions to other public health problems, such as obesity, is limited. The contributions of this paper may also increase the knowledge and understanding of the complexity of the work stress and obesity issues in manufacturing environments.

## 2. Literature Review

By way of background, this study explains the importance of work stress in its association with severe health damage. It also presents the concept of obesity and its measurement according to the WHO, the theoretical issues related to obesity and work stress, and the relevance of physical activity and its relationship with work stress and obesity. Finally, it provides the theoretical framework supporting the relationships between work, stress, and physical activity as well as the role of the latter in the prevention of both disorders.

### 2.1. Important Work Stress Effects on Human Health

Work stress is defined as the physical and emotional response to the damage created by the imbalance generated when the job requirements that an individual must meet exceed his/her available resources and capabilities [25]. In this sense, work stress occurs when the demands of the job do not match or exceed the worker’s capacities, resources, or needs [26]. Authors such as Calabrese report that the work pressure workers perceived from their jobs and the hours worked were decisive factors for the onset of stress [27]. Stressors can harm health by triggering harmful responses since they are negative stimuli [28]. In addition, they exert a negative impact on health through gradual and permanent wear of biological systems and can cause, among other physical diseases, psycho-emotional deterioration, behavioral disorders, and emotional disturbances [29].

One of the most widely recognized models in the study of occupational stress is Karasek’s demand–control model Job Content Questionnaire (JCQ) [24], which addresses dimensions such as work demands, physical demands, decision-making authority, co-worker support, supervisor support, skill use and job insecurity [24]. Because of the model’s relevance to this research, such dimensions are described in the following paragraphs.

The “Job demands” dimension is related to aspects such as ambiguity and conflict in work roles, employees’ perception of having to work excessively and rapidly, and the time pressure they undergo to finish the tasks [30]. It includes questions such as whether the completion of a job requires performing at a fast pace, facing higher degrees of difficulty or working excessively. It also implies the perception of having insufficient time to finish the tasks and feeling pressured by additional, conflicting demands from others. The “Job decision-making authority” dimension refers to the extent to which an individual can influence what happens in the workplace. The “Skill discretion” dimension addresses topics from a variety of tasks, whereas the “Social support” dimension refers to the support received by both supervisors and co-workers [31]. Finally, the “Job insecurity” dimension relates to the feeling of uncertainty of losing a job [32].

Additionally, studies have confirmed the existence of the relationship between work stress and physical activity and have shown that the latter may decrease the adverse effects of work stress. Several authors have recognized the importance of physical activity in preventing the damaging effects of work stress on human health. For example, the study by Nikell and Suarez mentions that good eating habits and physical activity are relevant factors in reducing stress. They establish that physical activity plays an important role since it can be considered a defense against work stress; thus, the more physically active people are, the less they will experience work stress-related problems [24]. Furthermore, another study by Rios mentions that the ability to adapt to stress increases when people are physically active [33].

### 2.2. Obesity and Work Stress

On the other hand, obesity is a public health problem found not only in developed nations but also in developing countries. The WHO considers obesity as an epidemic of a chronic non-communicable disease that begins at an early age and has multiple causes. It is defined as an abnormal or excessive accumulation of fat that can be harmful to health. The study of this problem has required the determination of proper measurements. Accordingly, one of the most widely recognized obesity-measuring indicators is BMI or body mass index. According to the WHO, a person with a BMI equal to or greater than 30 kg/m^2^ is considered obese, while one with a BMI equal to or greater than 25 kg/m^2^ is considered overweight. Obesity is also a risk factor for a variety of chronic illnesses, including diabetes, cardiovascular diseases, and cancer [1]. The data regarding obesity concerning the prevalence of this problem nationwide recently indicate that 39% of adults age 18 or older were overweight, and these figures are increasing yearly [34]. In addition, data from the National Health and Nutrition Survey evaluating 38,208 adults showed that 71.28% of the population, or the equivalent to 48.6 million people, featured obesity and overweight. Recently, this survey estimated 2.3 billion overweight people and 700 million obese people and affirm that even though multiple causes and risk factors have been related with this problem; one of the most widely accepted theory establishes that obesity is caused mainly by poor eating habits or by an imbalance between the calories consumed and the energy spent [34].

Currently, there is general agreement among researchers regarding the lack of studies establishing a clear relationship between work stress and obesity even despite the evidence pointing to the direct relationship between work stress and burnout [35]. In addition, Muñoz [36] recently mentioned that the relationship between work stress and BMI is not a new finding since many stressors produce anxiety towards food. On the other hand, a study on strategies to cope with stress mentions that different genetic, psychological, and social factors such as psychosocial stress have been associated with obesity and have been recognized to affect people’s quality of life [37,38]. In addition, Santana [39] presented results showing a relationship between being stressed and displaying eating behavior disorders, which is reflected in obesity and overweight.

The above phenomena constitute evidence of the need to increase knowledge about the problem in order to take effective preventive actions. This is the reason why some of our previous studies have explored the relationship between work stress and body mass index and their effect on Mexican managers. One study presents a descriptive study on Burnout [40]. Likewise, Armenta et al. and Macías et al. studies present the relationship between the dimensions of burnout and the BMI, concluding that additional factors are needed to better explain the BMI variable [41,42,43].

### 2.3. Theoretical Framework for the Relationship between Physical Activity, Work Stress, and Obesity

The importance of physical activity (PA) in preventing both obesity and work stress has also been documented. PA includes the practice of a sport, which is known to increase the quality of life and to contribute to people’s well-being and health improvement [44]. Thus, studying individuals’ physical activity is highly relevant to understand the alarmingly increasing frequency in obesity cases both in Mexico and globally. Furthermore, an active lifestyle with regular PA has positive effects on the control and prevention of chronic diseases such as obesity, hypertension, and type II diabetes mellitus.

On the other hand, on the psychological level, physical activity can also effect positive changes by helping to increase self-esteem and self-satisfaction, improve mood in general, and decrease anxiety and depression. A study by Ortiz and Gómez [45] discussed the important role that sports and physical activity play in the prevention of diseases such as obesity. Above all, the study lists the psychological benefits of PA on mental health, for example, lowering the frequency of stress symptoms. One additional study by Obando et al. [46] reports that it has been scientifically proven that physical activity, sports, and recreational activities contribute to reducing stress and improving overall health. Furthermore, Gallego et al. have investigated the relationship between physical activity and job content, affirming that physical activity contributes to the decrease of work stress and anxiety levels in people and recommending it as one of the most effective ways to decrease stress [47]. Obando endorses Gallego’s view on the importance of physical activity as it holds beneficial effects on health, and in this case, physical activity is one of the factors that significantly reduces work stress, thus increasing the overall health of those who engage in it.

In light of all of the above, this research establishes the following research questions:

1. What is the relationship between the work stress dimensions of the job content (JCQ), the physical activity dimensions, and BMI in obese (<30 kg/m^2^) middle and senior managers in the Mexican manufacturing?
2. What is the relationship between the physical activity dimensions and BMI in obese (<30 kg/m^2^) middle and senior managers from the Mexican manufacturing industry?
3. How is the relationship between work stress in the JCQ and physical activity associated with BMI in obese (<30 kg/m^2^) middle and senior managers from the Mexican manufacturing industry?

The general hypotheses are:

**Hypothesis** **1 (H1).** *There is a positive relationship between the work stress dimensions of the JCQ and BMI in obese (<30 kg/m^2^) middle and senior managers from the Mexican manufacturing industry.*

**Hypothesis** **2 (H2).** *There is a negative relationship between the physical activity dimensions and BMI in obese (<30 kg/m^2^) middle and senior managers from the Mexican manufacturing industry.*

**Hypothesis** **3 (H3).** *H3: There is a negative relationship between the work stress of the JCQ and physical activity towards BMI in obese (<30 kg/m^2^) middle and senior managers from the Mexican manufacturing industry.*

**Hypothesis** **4 (H4).** *H4: The above hypotheses establish that there will be a positive relationship between Job Content and BMI, and a negative relationship between physical activity and BMI with the corresponding effects.*

These hypotheses will be theoretically supported and tested in the following sections of this paper.

## 3. Materials and Methods

The aim of this research will be achieved through three structural equation models. The first will determine the relationships between the dimensions of the work stress by JCQ and BMI. The second will determine the relationship between the dimensions of the Baecke’s physical activity questionnaire and BMI. Finally, the third integrative model will determine the relationship between the significant dimensions of both work stress by JCQ and physical activity and BMI.

### 3.1. Participants and Characteristics of the Sample

The study was conducted in 16 manufacturing companies in Ciudad Juarez, Chihuahua, Mexico from November through May 2019. The duration of the study was of three months, as sufficient time was needed to collect and debug data; the analytical process required approximately one month. A sample of 255 staff in middle and higher management from the maquiladora industry participated in this study. Their age range was 31–40 years, and 67.1% of the workers were male and 32.9% were female. Regarding their schooling, 55.7% held a bachelor’s degree, 23.7% had finished high school studies only, 13.2% had a master’s degree, 6.6% had only finished middle school, 0.6% had a Ph.D., and 0.1% had only finished elementary school. As for their marital status, 55.4% were married, 30.1% single, 8.4% lived in free union, 5.3% were divorced, and 0.9% were widowed. According to the hours worked per week, 52.5% worked a total of 45 h a week, 26.9% worked 48 h, 12.8% worked 42 h, and 7.8% worked 52 h or more a week. The information regarding their current position and categorization showed the following composition: Managers made up 9.9% of the sample, supervisors 25.3%, technicians 25.9%, group leaders 11.3%, and others such as administrative staff 27.7%.

This section offers a summary of statistics regarding the percentage of managers who suffer from work stress and the percentage of managers who suffer from obesity to any degree. Additionally, a sedentary criterion was found for the physical activity indices.

In the case of work stress, 25% of managers show evidence of suffering from it according to the JCQ Job Strain Index [48].

Concerning the prevalence of obesity among managers, 72.94% of them feature first degree obesity, 17.64% show it in second degree, and 9.41% of the participants suffer from the third degree type.

In reference to the physical activity, indices obtained from managers offered the diagnosis of a sedentary lifestyle in all three dimensions (physical sports activity, physical activity at work, and physical activity in leisure time). Interpretation of these indices and reference values were available from Baecke et al. [49]. As abbreviations, these variables were renamed (sports, work and leisure-time), respectively, in the structural models.

### 3.2. Materials

The instruments of Job Content Questionnaire [48] in its Spanish version and Baecke’s short Physical Activity Questionnaire [49] were used; lastly, a sociodemographic factors questionnaire was utilized.

#### 3.2.1. Job Content Questionnaire

Cedillo [50] created the Spanish version of the instrument used in this research that consists of 27 items divided into 6 dimensions (job demands, supervisor support, co-workers support, skill discretions, decision-making authority, and job insecurity.) The instrument is answered by means of a Likert scale with different values. The reliability of the questionnaire in our sample was checked using Cronbach’s alpha and the result was statistically reliable with a value of 0.827.

#### 3.2.2. Baecke’s Physical Activity Questionnaire

Created by Baecke [49], this instrument makes it possible to obtain physical activity indices in different dimensions such as work, sports and leisure time. The questionnaire for measuring physical activity consists of 23 questions; one of these questions leads to other questions to obtain more information about the dimension being assessed. Questions are answered by means of a Likert scale with different values for each item. The results presented in this questionnaire are the physical activity index at work, the physical activity index in sports, and the physical activity index during leisure time; such indices are then calculated using equations pre-established by the author. The reliability of the instrument for this specific sample was verified using Cronbach’s alpha, and the result was statistically acceptable as it featured the value of 0.774.

#### 3.2.3. The Sociodemographic Data Questionnaire

This questionnaire was designed to obtain more information about the characteristics of the sample. It features sociodemographic data such as age, gender, marital status, current job position, seniority in the company, hours worked per week, and other data such as weight and height, which were obtained by companies’ medical staff.

### 3.3. Methods

#### 3.3.1. Sample Identification

A non-probabilistic convenience snowball sampling was used for this study. The researchers presented the project to the members of the medical committee from the manufacturing sector. Those companies that agree to participate were visited to collect data. Several factors were considered to identify the sample for this research; first, the need found in the literature regarding the effects of work stress and Job Content on employees of the manufacturing industry, and then the concern about the obesity grades in middle and upper management in this same industry. It is also necessary to highlight that the people occupying these job positions are key to the development and performance of the organization, a reason why their physical and mental health is relevant. In addition, work stress and limited physical activity may lead to poor performance; they may increase the risk of developing chronic diseases, which increases costs due to losses in productivity and medical expenses [51,52]. On the other hand, the reason this sample was studied in Ciudad Juarez is the location’s relevance as one of the top three industrial cities in Mexico and the seventh largest manufacturing center of North America. The city hosts 327 manufacturing “*maquiladora”* industries with over 300,000 direct and indirect employees [53]. Additionally, this Ciudad Juarez, Chihuahua, Mexico-El Paso Texas, United States of America (USA) border region offers a unique geographic point that gives direct entry to the USA market and represents the largest bi-national urban zone in North America.

#### 3.3.2. Database Debugging

At this stage of data processing, descriptive analyses are performed to detect lost values and outliers. Since ordinal values are used (Likert scale) mostly for the questionnaires, the lost values should be replaced by the median; however, Hair [54] points out that the amount of replaced values should not exceed 10% of total data per variable. As for the weight and height, the lost values can be replaced by the mean value as that is a nominal scale. Subsequently, the outliers should be analyzed through box charts using software IBM Statistics SPSS 21^®^ (IBM Software Group, Attention: Licensing, 233 S. Wacker Dr., Chicago, IL 60606, USA). This procedure helps reduce errors caused by deficient data input or observation.

#### 3.3.3. Generation and Validation of the Structural Equation Models

At this stage of the methodology, structural equation models were generated and validated. Likewise, the following variables were stated for these models: in terms of the JCQ, the work control dimensions were skill discretion, decision-making authority, and social support (coworkers support and supervisor support). In reference to job demand dimensions, the variables stated were job demands and job insecurity; in terms of physical activity, the considered variables were work physical activity, sport physical activity and leisure-time physical activity; and, finally, in terms of BMI (BMI >= 30 kg/m^2^). The relationships of the dimensions raised in the research hypotheses were analyzed by means of the WarpPLS 6.0^®^ (ScriptWarp Systems P.O. Box 452428 Laredo, Texas, 78045 USA) software, and the criterion for the acceptance or rejection of the hypotheses was based on the *p*-values.

#### 3.3.4. Direct Effects

Direct effects were obtained for each established model and for the participating latent variables. The direct effects are those given per segment from one (latent) variable to another and in which the hypotheses posed are validated. The directs effects are presented and explained for each model separately in the results section.

#### 3.3.5. Model Efficiency Indices

The proposed models are analyzed using the following general efficiency indices provided by WarpPLS 6.0^®^ after processing the hypothetical model. Kock [55] highlights the following indices: average path coefficient (APC), average R-square (ARS), average adjusted R-square (AARS), average block variance inflation factor (AVIF), average full collinearity (AFVIF), Tenenhaus goodness of fit (GoF), Simpson’s paradox (SPR), the R square contribution ratio (RSCR) and the statistical suppression ratio (SSR). Model efficiency indices are presented and explained for each model separately in the results section.

#### 3.3.6. Coefficients of Latent Variables

The coefficients used to validate the questionnaires are the values of R square, Q square, adjusted R square, composite reliability coefficient, Cronbach’s alpha, and the VIF and AFVIF values for latent variables. Coefficients of latent variables are presented and explained for each model separately in the results section.

#### 3.3.7. Sum of the Model’s Total Effects

In this step, the values that made up the regression value of R^2^ of each dimension were analyzed as was the case, this was to analyze what percentage of each variable explained the other. Indirect effects were obtained through other latent dimensions or variables with two or more segment paths. The sum of these effects yielded the total effects or sum of total effects. The sum of total effects is the sum of both direct and indirect effects among the latent variables.

## 4. Results

### 4.1. Results of Model 1: Relationship between the Dimensions of the Job Content and BMI

**Figure 1:**
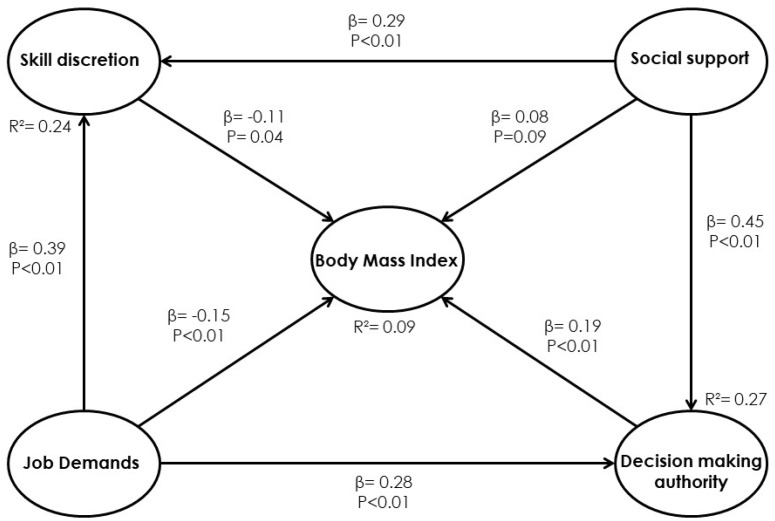
Model 1. Relationship between the job content dimensions and body mass index (BMI).

The relationships between the JCQ dimensions and BMI in the obese managers’ sample are proposed. Each of the paths indicates the values of the β parameters and the *p*-values. Regarding the first one, the results in Figure 1 show that more than 85% of the established relationships are statistically significant. Thus, the skill discretion reduces the BMI variable (β = −0.11; *p* = 0.04), the job demands reduce BMI (β = −0.15; *p* < 0.01), and the decision-making authority increases BMI (β = 0.19, *p* < 0.01), the case being different for the social support dimension l.

**Figure 2:**
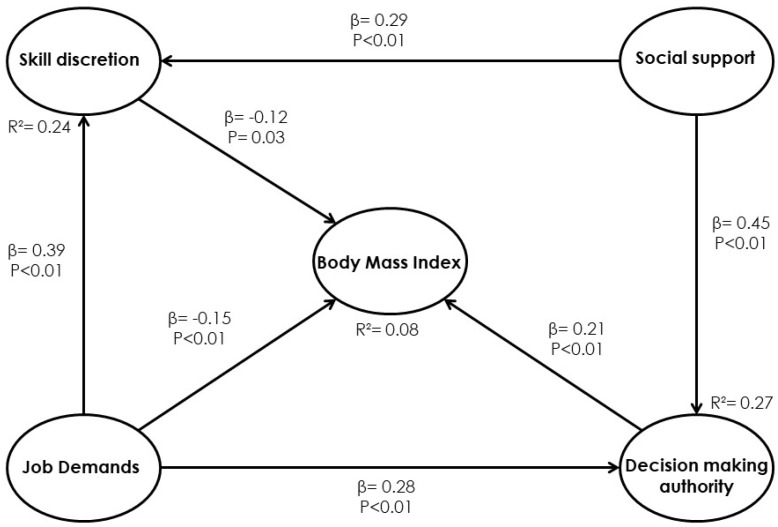
Final Model 1 for the relationships between the Job Content Questionnaire (JCQ) dimensions and BMI.

Once, an iteration of the model was conducted by means of the software analysis with the aim of removing the social support dimension that was not statistically significant; the model’s result is shown in Figure 2.

Hypotheses and structural equations for the effects of JCQ dimensions on BMI in the model are established as follows:

**Hypothesis** **1 (H1).** *There is a negative relationship between the skill discretion variable and BMI in obese (<30 kg/m^2^) middle and senior managers from the Mexican manufacturing industry.*

**Hypothesis** **2 (H2).** *There is a positive relationship between the social support variable and BMI in obese (<30 kg/m^2^) middle and senior managers from the Mexican manufacturing industry*

**Hypothesis** **3 (H3).** *There is a negative relationship between the job demands variable and BMI in obese (<30 kg/m^2^) middle and senior managers from the Mexican manufacturing industry*

**Hypothesis** **4 (H4).** *There is a positive relationship between the decision-making authority variable and BMI in obese (< 30 kg/m^2^) middle and senior managers from the Mexican manufacturing industry.*

BMI of obese managers’ sample = −0.12 × skill discretion − 0.15 × job demands + 0.21 × decision-making authority + Error (1)

Equation (1) shows the direct effects of the dimensions of the job content dimensions on BMI in the obese managers’ sample. The equation explains the contribution of the beta (β) value of each latent variable to the dependent variable.

#### 4.1.1. Model 1. Direct Effects

The aim of Figure 2 and Table 1 is to show relationships and the direct effects of the latent variables. Every direct effect is associated with a beta (β) value and a *p*-value. The former indicates dependency in standard deviations, whereas *p* is the significance value from the hypothesis test. Significant relationships have *p*-values lower than 0.05, and, consequently, they are statistically significant at a 95% confidence level on the direct effect results that feature the statistically significant negative direct effect of the skill discretion (*p* = 0.03) and job demand (*p* < 0.01) dimensions on BMI, as well as the statistically significant positive direct effect of the decision-making authority dimension on BMI. Thus, the results indicate that when the skill discretion increases by a standard deviation, BMI will decrease by 0.12 units or 12%; when the job demands dimension increases by a standard deviation, BMI will decrease by 15%; and when the decision-making authority increases by a standard deviation, BMI will increase by 0.21 units or 21%. Similarly, job demands contributes to the increase in the skill discretion by 39% (*p* < 0.01) and decision-making authority by 0.28 units (*p* < 0.01). The social support dimension has no direct effects on BMI, but it contributes to the increase in the skill discretion by 29% (*p* = 0.01) and the decision-making authority by 45% (*p* < 0.01).

#### 4.1.2. Model 1. Efficiency Indices 

The purpose of Table 2 is to present the results of the efficiency indices of Model 1. The efficiency indices of APC, ARS and AARS show *p*-values < 0.05. Accordingly, the model is efficient and holds predictive capacity, and that, on average, all the parameters measuring the relationships between the latent variables are statistically significant at a 95% confidence level. This is also due to the fact that the values are lower than 0.05, the value recommended by Kock [55]. For the AVIF and AFVIF values, figures lower than 3.3 were obtained, collinearity problems being thus ruled out. On the other hand, the GoF indicator is 0.340, which attributes large explanatory power to the model [56]. The SPR value equals 1, which indicates that at least 70% of the road routes in the model are free of paradox. In addition, the fact that the RSCR value equals 1 indicates that the model is free of negative contributions from R^2^ (since the independent latent variable reduces the percentage of variance explained), which occurs along with the Simpson paradox. Finally, the SSR value, which is >0.7, indicates that at least 70% of the paths in the model is free of statistical suppression.

#### 4.1.3. Model 1. Latent Variable Coefficients

Table 3 aims to show the latent variable coefficients for Model 1. As it can be observed, the Cronbach’s alpha and compound reliability values higher than 0.7 are acceptable, except for the job demands variable and skill discretion variable. In these cases, lower values can be acceptable for exploratory studies as it is in this research [56].

Similarly, because all VIF values are lower than 3.3, these results indicate that there are no collinearity problems between the latent variables. Finally, all Q^2^ values appear to be greater than 0 and like their corresponding R^2^ values. These results confirm that the latent variables have acceptable parametric validity.

### 4.2. Results of Model 2: Relationship between the Physical Activity Dimensions and BMI

**Figure 3:**
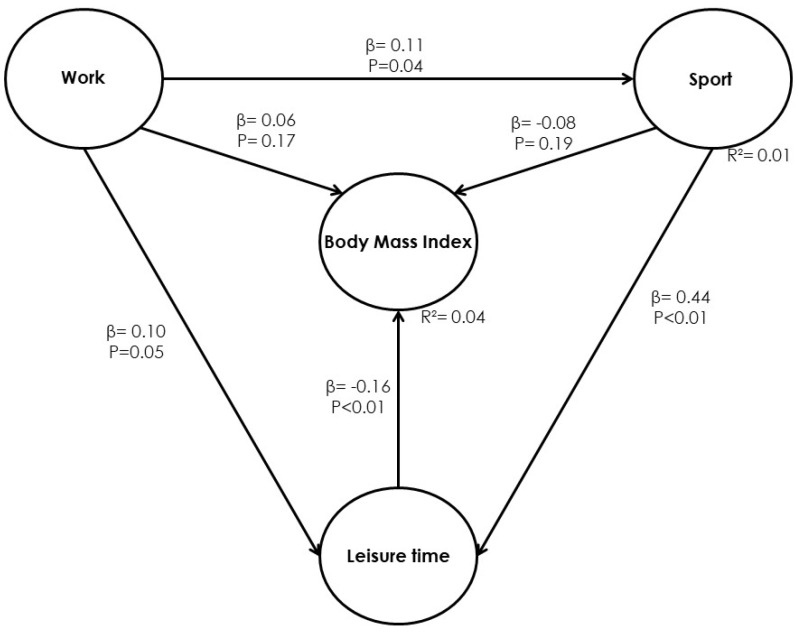
Model 2. Relationships between physical activity dimensions and BMI.

The structural model, 2, is presented in Figure 3. It shows that only 50% of the established relationships are statistically significant. Thus, the during leisure-time (leisure) index reduces BMI β = −0.16 (*p* < 0.01). This is not the case for the dimensions of physical activity at work (work) index and physical activity at sports (sport) index, as shown in Table 4. Due to the above, it was necessary to perform one iteration to remove these relationships from the model and observed their behavior.

Hypotheses and the structural equation model for the relationships between physical activity and BMI in the obese managers’ sample are established as follows:

**Hypothesis** **1 (H1).** *There is a negative relationship between the sports activity and BMI in obese (<30 kg/m^2^) middle and senior managers from the Mexican manufacturing industry.*

**Hypothesis** **2 (H2).** *There is a positive relationship between the work activity variable and BMI in obese (<30 kg/m^2^) middle and senior managers from the Mexican manufacturing industry.*

**Hypothesis** **3 (H3).** *There is a negative relationship between the leisure-time variable and BMI in obese (<30 kg/m^2^) middle and senior managers from the Mexican manufacturing industry.*

#### 4.2.1. Model 2. Direct Effects 

**Figure 4:**
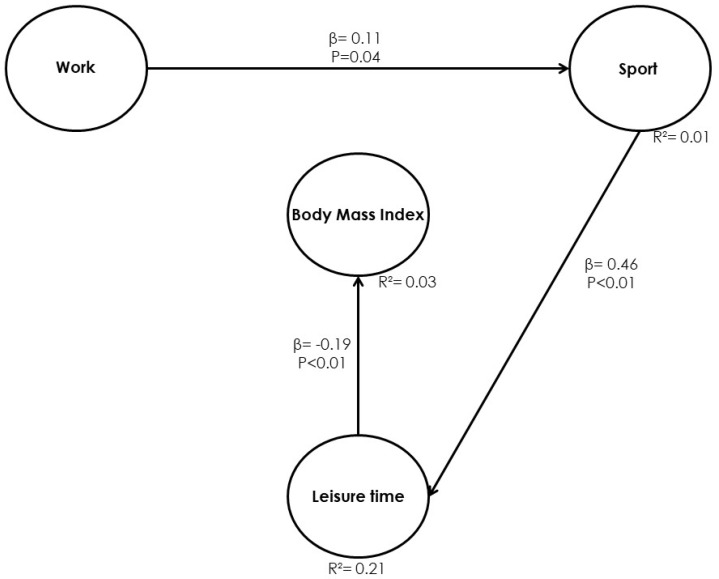
Final Model 2 for the relationships between the physical activity dimensions and BMI.

The purpose of Table and Figure 4 is to present the direct effects of Model 2. The statistically significant negative direct effect of the leisure dimension (*p* < 0.01) with respect to BMI is displayed. Thus, it is inferred that when the leisure-time variable (leisure) increases by one standard deviation, BMI will decrease by 0.19 units. The work variable (physical activity at work) and sport variable (sports physical activity) have no direct effects on BMI, but physical activity at work contributes to increasing the sports physical activity by 0.11 units (*p* = 0.04) and the latter contributes to increasing the leisure-time variable by 0.46 units (*p* < 0.01).

BMI of obese managers’ sample = −0.19 × leisure-time + Error(2)

Equation (2) shows the direct effects on BMI in the obese managers’ sample and the unique significant dimension of leisure-time physical activity. The equation explains the contribution of the beta of each latent variable to the dependent variable.

#### 4.2.2. Model 2. Model Efficiency Indices

The purpose of Table 5 is to present the results of the efficiency indices of Model 2 shown in Figure 4. The three efficiency indices of APC, ARS, and AARS present *p*-values < 0.05; from this, it can be determined that the model is efficient and holds predictive capacity, and that all the parameters that measure the relationships between the latent variables are statistically significant at a 95% confidence level. This is also true because the values are lower than 0.05, the recommended value [56]. The values obtained for AVIF and AFVIF were lower than 3.3, which eliminates collinearity problems. On the other hand, the GoF indicator is 0.230, which causes the model to have little explanatory power [56]. The fact that the SPR value equals 1 indicates that at least 70% of the road routes in the model are paradox-free. The RSCR value of 1 indicates that the model is free of negative contributions from R^2^ (the independent latent variable reduces the explained variance percentage), which occurs along with the Simpson’s paradox. Finally, an SSR value > 0.7 indicates that at least 70% of the percentage of paths in the model is free of statistical suppression.

#### 4.2.3. Model 2 Latent Variable Coefficients

The latent variables coefficient indices shown in Table 6 indicate that the model is generally efficient and can be used to interpret the relationships between the physical activity and BMI dimensions. For the BMI and leisure variables, the values of square R and adjusted square R are greater than 0.02. Therefore, from a parametric point of view, those two dependent latent variables or explained by an independent latent variable have enough predictive validity. In addition, the square Q values of all variables are greater than 0 and are close to their respective squared R and adjusted squared R. This indicates that each block of latent variables in the model is associated with the criterion variable (BMI). As the internal validity values, all the reliability values of the alpha and Cronbach compounds are greater than or very close to 0.7, except for the leisure-time variable. In this case, those Cronbach’s alphas value lower than the reference accepted value (0.7) could be considered acceptable for exploratory studies, which indicates that the latent variables feature good reliability and that the items or questions are associated with each of the dimensions presented [56]. In this manner, the variables present internal validity values. Besides, regarding the AVE analysis, all values are above 0.5, the value recommended by Fornell and Larcker [56], except for the physical activity at work (work). However, this value can be acceptable since this variable is not directly related to the BMI [57]. This indicates that all latent variables have convergent validity. Finally, all the variables have adequate collinearity (VIF), since all of them are lower than 3.3, the maximum value allowed. Therefore, based on the results of the analysis of these parameters, the structural equation model (SEM) can be considered adequate and can be analyzed.

### 4.3. Results of Model 3: Effects of Physical Activity and Job Content (Work Stress by the JCQ) with BMI

**Figure 5:**
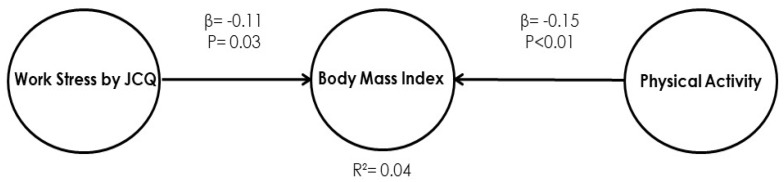
Model 3. Relationships between physical activity, job content (work stress by JCQ), and BMI.

The purpose of Figure 5 is to display the integrating model relating the three main constructs of the problem, namely the physical activity, job content (work stress by JCQ), and BMI of obese managers. This model is created from the significant relations established in the previous models (Model 1 and Model 2).

The hypotheses and structural equation model for the relationships of the physical activity variable and job content (work stress by JCQ) variable on BMI in the obese managers’ sample are established as follows:

**Hypothesis** **1 (H1).** *There is a negative relationship between the job content (work stress by JCQ) variable and BMI in obese (<30 kg/m^2^) middle and senior managers from the Mexican manufacturing industry.*

**Hypothesis** **2 (H2).** *There is a negative relationship between the physical activity variable and BMI in obese (<30 kg/m^2^) middle and senior managers from the Mexican manufacturing industry.*

BMI of obese managers’ sample = −0.113 × work stress by JCQ − 0.146 × Physical Activity + Error(3)

Equation (3) shows the direct effects of the job content variable (work stress by JCQ) and the physical activity variable on BMI in the obese managers’ sample. The equation explains the contribution of the beta of each latent variable to the dependent variable.

#### 4.3.1. Model 3. Direct Effects

From the direct effect results shown in Figure 5 and Table 7, it can be observed that all relationships are statistically significant with a 95% confidence level. Thus, when the job content (work stress by JCQ) increases by one standard deviation, BMI is reduced by 0.113 units (*p* = 0.03); and, when the physical activity variable increases by one standard deviation, BMI is reduced by 0.15 units (*p* = 0.01).

#### 4.3.2. Model 3. Efficiency Indices

The purpose of Table 8 is to present the Model 3 efficiency indices and demonstrates that the model of the relationship of physical activity, Job Content (work stress by JCQ) and BMI is efficient since all the *p*-values are lower than 0.05. Likewise, the results prove that the model has good predictive power in all its parameters and good explanatory power, the latter of which is according to the GoF index. In conclusion, the model is efficient.

#### 4.3.3. Model 3. Latent Variable Coefficients

Table 9 presents the latent variable coefficient indices. Hence, these indicate that the model is generally efficient and can be used to interpret the significant relationships in the previous models in relation to BMI in a general model. The adjusted-R and R-square values are greater than 0.02. Therefore, from a parametric point of view, all dependent latent variables or those explained by an independent latent variable hold enough predictive validity. In addition, the Q^2^ values of all variables are greater than 0 and are close to their respective R and R^2^ value. This indicates that each latent variable block in the model is associated with the criterion variable. Regarding the internal validity values, the Cronbach’s alpha and compound reliability values are greater than 0.7, except for the physical activity variable. In this case, those Cronbach’s alphas value lower than the reference accepted value (0.7) can be considered acceptable for exploratory studies, which indicates that the latent variables feature good reliability and that the items or questions are associated with each of the dimensions presented [56]. In this manner, the variables present internal validity values. The AVE analysis shows that the BMI variable is the only one whose value is above 0.5, the one recommended by Fornell and Larcker [56]. Finally, all variables hold adequate collinearity (VIFs), since none of them feature values greater than 3.3, the maximum value admitted. Therefore, based on the results of these parameters analysis, it can be inferred that the SEM is adequate and can be analyzed.

## 5. Discussion

The results of the first two models show significant relationships between the skill discretion and job demands dimensions contained in the job content variable and BMI, as well as between the leisure-time physical activity contained in the physical activity variable and BMI. Lastly, the results of the integrative model infer that even work stress and physical activity have direct negative effects on BMI.

The first model relates the Job Content dimensions (work stress by JCQ) with BMI, its results showing that three of the four JCQ dimensions used were statistically significant. The variable with the highest explanatory power over BMI is decision-making authority, which can mean that when managers feel limited regarding the job decisions they can make, they become hesitant towards how to perform it; similarly, when they feel that their opinions at work are neglected, their stressful perceptions show an impact on their BMI. Thus, when this variable (decision making-authority) increases by one unit, BMI increases by 0.21 units.

On the other hand, the study found that the skill-discretion dimension is inversely related to BMI. This could be associated with the fact that when workers are able to use their learned skills and abilities, their sense of control regarding their job increases. This increase in the sense of control is a variable that may reduce the feeling of stress. Considering that managers tend to have stable characteristics (unless some disorder is present), a greater sense of control could result in healthier behavior, which would have an impact on BMI. Regarding the demand scale, a negative relationship with BMI was found, reflecting that the greater the work demands, the lower the BMI; this means that these employees’ workload can lead to a lower BMI. This is an interesting aspect since stress is associated with physiological activation, which would normally result in a lower BMI than that of workers with fewer demands. Thus, these results lead us to agree with Muñoz [36] and Santana’s [39] studies, which show the relationship between stress and BMI, except that in our case the sample comprises middle and upper managers featuring obesity.

Conversely, the second model on physical activity and BMI showed interesting results since the physical activity during the leisure-time dimension was the only variable directly related to BMI. Thus, reduced physical activity, or sedentarism, outside of work contributes to the increase in BMI as described by Quirantes’ study [57], in whose sample most of the obese people led a sedentary lifestyle. The rest of Baecke’s questionnaire dimensions were not significant. In this regard, it can be confirmed that employees’ work demands have resulted in long work hours and practically non-existing physical activity, both of which show a relationship with BMI. That means that the variable of physical activity at work (work) is of no relevance when explaining BMI. However, this study found that physical activity during leisure-time (leisure) is the only variable significantly related in a direct though inverse way with BMI. Perhaps an increase in these workers’ physical activity during their working hours would result in a significant relationship with BMI. Therefore, it would be important to increase physical activity during the working day by instructing workers to change their posture several times during the workday as well as implementing physical activity through workplace gymnastics or opening gym facilities in the workplace to positively influence the reduction of BMI, especially in obese individuals. The promotion of physical activity at work is an essential, although not unique, aspect of the increase in energy consumption among workers with sedentary work.

Finally, the integrative model shows the relationship between physical activity, work stress by job content, and BMI among obese managers in the Mexican manufacturing industry. Although the model features a relatively low explanation, this relationship is indeed a significant one. In this regard, it must be considered that there are multiple factors influencing BMI prediction (i.e., genetic, biological, physiological, etc.). However, few studies analyze the role of collective aspects (such as work stress) in BMI as an obesity indicator. These results, then, support the scarce literature found, as is the case with the study by Azofeifa et al. [58], which finds that the greater the lack of physical activity, the higher the level of stress that a person can show. In this study, although variables such as work stress or physical activity offer but a small explanation of the percentage of BMI variance, they are still considered relevant. The effects of work-stress on BMI have been generally ignored, especially among high and middle managers, even though, according to the results obtained in this study, those factors do seem to be directly related to it. In this sense, considering that, for managerial positions, exposure to work stress is habitual and long-lasting, it should be highlighted that addressing stress itself could be a beneficial factor for these workers’ health.

This work is theoretically supported by previous research that has studied some of these problems, as well as their dimensions. In turn, it provides the theoretical framework to support the hypotheses of models that confirm to some extent the relationships between job stress, physical activity and BMI among obese managers. In addition, the industrial sector would benefit from the awareness that serious diseases such as type II diabetes, hypertension, cholesterol, fatty liver, coronary, vascular and respiratory diseases and even some types of cancer may be associated with obesity. In general, a low-calorie diet is recommended for obese patients. Patients suffering from occupational stress generally have a high intake of saturated fats, sugars and carbohydrates, and this affects their health and contributes to the generation of these chronic diseases [59,60]. Therefore, an additional recommendation is to promote good eating habits among patients with obesity and fatty liver problems: a diet consisting of fruits, vegetables, vitamins (C, D and E), some foods, such as fish containing Omega 3 and olive oil for cooking, which is a healthier option. These and other foods help fight and reduce the above-mentioned diseases [61].

Additionally, it should be mentioned that this research has been financially supported to study the relationship between work stress and obesity among the vulnerable and commonly disregarded population of Mexico’s middle and higher management. The results and analyses of such a complex problem, considering its different variables and aspects, have been studied in our previous work. Consequently, the results earlier obtained led us to conclude that other factors must be included in the study to better explain the relationship work stress, obesity and/or overweight since the explanatory power found by means of the structural models developed was very low. Therefore, the scope of this research was expanded to include physical activity as a factor that may help explain the variation of BMI variable as an indicator of obesity and overweight among Mexican managers.

The contribution of this work lies in the lack of studies that relate such variables together to explore the relationship among work stress by job content dimensions, physical activity, and BMI in Mexican managers specifically.

Regarding this study’s limitations, it can be said that it has included important variables that explain the variation in BMI, for example, physical activity, while other factors that could help describe BMI have been excluded. Consequently, trying to cover other factors might help explain BMI. Another limitation relates to the different instruments that can be used to measure stress and physical activity since this research used only one instrument for each variable. Therefore, it would be interesting to obtain closer day-to-day measures (by diaries, for instance), of both work stress and physical activity, which might allow for the observation of workers’ behavior on a regular basis.

## 6. Conclusions

This research aimed to determine the relationship and effects of job content and physical activity on BMI among obese managers of the Mexican manufacturing industry.

Accordingly, the conclusions of the first model about the relationship between JCQ dimensions and BMI in the obese managers’ sample is that the four dimensions involved are statistically significant, three directly and one indirectly. Thus, the skill-discretion dimension is directly significant with a β = −0.12 and a *p*-value of 0.03, and, through this dimension, the social support is indirectly significant. Additionally, the job demands variable is directly significant, showing a β = −0.15 and a *p*-value < 0.01; and, finally the decision-making authority variable is directly significant with a β = 0.21 and a *p*-value < 0.01. Additionally, the explanatory power of the model is medium and corresponds to an R^2^ = 0.08 value.

In relation to the second model, conclusions of the relationships between the physical activity dimensions and BMI in the obese managers’ sample, are that the three dimensions of the physical activity questionnaire are significant, although only the leisure-time variable is directly significant, showing a value of β = −0.19 and a value of *p* < 0.01. While physical activity at work and sport physical activity are both indirectly significant, the three variables manage to obtain a medium explanatory power of the model and correspond to an R^2^ = 0.03.

Finally, the conclusions of the integrating model, between job content (work stress by JCQ), physical activity, and BMI in the obese managers’ sample are that both the variable of work stress by JCQ and the variable of physical activity are significantly related to BMI. In the case of the JCQ work stress variable, the variable has a β = −0.11 and a *p*-value of 0.03; and the variable of physical activity presents a β = −0.15 and a *p*-value < 0.01, resulting in low explanatory power and correspond to and R^2^ = 0.04.

### 6.1. Industrial Implications

Recently, in an attempt to decrease work stress in Mexico, the Mexican government has issued new occupational health regulations for the industrial sector. These are related to the psychosocial risks at work, where the topic of work stress is included. This is where the relevance of this research can be highlighted, especially when occupational health problems suffered by middle and high managers of the manufacturing industry are studied. These important job positions are usually underestimated and have been insufficiently addressed by the available literature. Additionally, obesity is considered a national priority problem in Mexico since the country’s population occupies the top positions in this health problem worldwide. Consequently, knowledge about the relationship between work stress and obesity may offer organizations a new perspective on how to manage both of these problems effectively. Companies around the world could develop strategies to increase and promote employees’ engagement in physical activity at work and to increase the practice of sports activities, especially by middle and higher managers. Formal organizational programs fostering healthy habits, psychological assistance, and work stress management techniques at work are also common practices among world-class companies pursuing high performance based on their employees’ wellbeing.

The results of this research help expand the knowledge about these occupational health problems in the industrial manufacturing sector. Such knowledge can be used to inform and encourage companies to intensify their attention to health-related problems, not only physical health but also mental wellbeing. Additionally, companies may increase their interest in the consequences of work stress and encourage aid through proposals for stress-coping strategies as well as human and organizational development to prevent these health problems in the light of new approaches.

### 6.2. Future Research

The results of this research pave the way for new causal research into overweight and obesity. As future work, we will seek to integrate other factors into the models, including eating habits and sociodemographic considerations, in addition to including other jobs, such as manufacturing operators. The objective of refining the models would be to provide literacy and a clearer understanding of the variance of BMI that can be related to obesity. Thus, it is intended to extend our research by developing software and cognitive systems to obtain knowledge and support the diagnoses and effective management on these issues in industrial environments.

## Figures and Tables

**Table 1:** Model 1. Direct effects of the relationships between JCQ dimensions and BMI.

To/From	Skill Discretion	Social Support	Job Demands	Decision-Making Authority
BMI	β = −0.12 (*p* = 0.03)		β = −0.15 (*p* < 0.01)	β = 0.21 (*p* < 0.01)
Skill discretion		β = 0.29 (*p* < 0.01)	β = 0.39 (*p* < 0.01)	
Decision-making authority		β = 0.45 (*p* < 0.01)	β = 0.28 (*p* < 0.01)	

**Table 2:** Model 1. Efficiency indices of the relationships between JCQ dimensions and BMI.

Indices	Value	Decision Criteria
Average path coefficient (APC)	0.269	*p* < 0.001
Average R-squared (ARS)	0.198	*p* < 0.001
Average adjusted R-squared (AARS)	0.190	*p* < 0.001
Average block (AVIF)	1.010	Acceptable <= 5, ideal <= 3.3
Average full collinearity VIF (AFVIF)	1.466	Acceptable <= 5, ideal <= 3.3
Tenenhaus (GoF)	0.340	small >= 0.1, medium >= 0.25, large >= 0.36
Sympson’s paradox ratio (SPR)	1.000	Acceptable >= 0.7, ideal = 1
R-squared contribution ratio (RSCR)	1.000	Acceptable >= 0.9, ideal = 1
Statistical suppression ratio (SSR)	0.857	Acceptable >= 0.7

**Table 3:** Model 1. Latent variable coefficients for relationships between JCQ dimensions and BMI.

	BMI	Skill Discretion	Social Support	Job Demands	Decision-Making Authority
R-squared	0.079	0.239			0.274
R-squared adjusted	0.068	0.233			0.269
Composite reliability	1.000	0.785	0.906	0.663	0.847
Cronbach’s alpha	1.000	0.673	0.880	0.493	0.729
Avg.var. extrac. (AVE)	1.000	0.405	0.329	0.550	0.649
Full collinearity. (VIFs)	1.011	1.917	1.192	1.252	1.956
Q-squared	0.078	0.241			0.275

**Table 4:** Model 2. Direct effects of the relationships between physical activity and BMI.

To/From	Physical Activity at Work (Work)	Sports Physical Activity (Sport)	Leisure-Time Physical Activity (Leisure)
**BMI**			β = −0.19 (*p* < 0.01)
**Sport**	β = 0.11 (*p* = 0.04)		
**Leisure-time**		β = 0.46 (*p* < 0.01)	

**Table 5:** Model 2. Efficiency indices of the relationships between physical activity dimensions and BMI.

Indices	Value	Decision Criteria
Average path coefficient (APC)	0.253	*p* < 0.001
Average R-squared (ARS)	0.087	*p* = 0.040
Average adjusted R-squared (AARS)	0.083	*p* = 0.045
Average block (AVIF)	No available	Acceptable <= 5, ideal <= 3.3
Average full collinearity VIF (AFVIF)	1.073	Acceptable <= 5, ideal <= 3.3
Tenenhaus (GoF)	0.230	small >= 0.1, medium >= 0.25, large >= 0.36
Sympson’s paradox ratio (SPR)	1.000	Acceptable >= 0.7, ideal = 1
R-squared contribution ratio (RSCR)	1.000	Acceptable >= 0.9, ideal = 1
Statistical suppression ratio (SSR)	1.000	Acceptable >= 0.7

**Table 6:** Model 2. Latent variables’ coefficients for the relationships between physical activity dimensions and BMI.

	BMI	Work	Sport	Leisure-Time
**R-squared**	0.034		0.012	0.214
**R** **-squared adjusted**	0.030		0.008	0.211
**Composite reliability**	1.000	0.818	0.907	0.760
**Cronbach’s alpha**	1.000	0.740	0.883	0.527
**Avg. var. extrac. (AVE)**	1.000	0.402	0.507	0.516
**Full collinearity (VIFs)**	1.009	1.027	1.118	1.139
**Q-squared**	0.034		0.017	0.183

**Table 7:** Model 3. Direct effects of the relationships between work stress, physical activity, and BMI.

	Job Content (Work Stress by JCQ)	Physical Activity
**Body Mass Index**	β = −0.113 (*p* = 0.033)	β = −0.15 (*p* = 0.009)

**Table 8:** Model 3. Efficiency indices for the relationships between work stress, physical activity, and BMI.

Indices	Value	Decision Criteria
Average path coefficient (APC)	0.130	*p* = 0.009
Average R-squared (ARS)	0.037	*p* = 0.138
Average adjusted R-squared (AARS)	0.029	*p* = 0.160
Average block (AVIF)	1.007	Acceptable <= 5, ideal <= 3.3
Average full collinearity VIF (AFVIF)	1.012	Acceptable <= 5, ideal <= 3.3
Tenenhaus (GoF)	0.143	small >= 0.1, medium >= 0.25, large >= 0.36
Sympson’s paradox ratio (SPR)	1.000	Acceptable >= 0.7, ideal = 1
R-squared contribution ratio (RSCR)	1.000	Acceptable >= 0.9, ideal = 1
Statistical suppression ratio (SSR)	1.000	Acceptable >= 0.7

**Table 9:** Model 3. Latent variable coefficients for the relationships between work stress, physical activity, and BMI.

	BMI	JCQ	Physical Activity
**R-squared**	0.037		
**R** **-squared adjusted**	0.029		
**Composite reliability**	1.000	0.826	0.584
**Cronbach’s alpha**	1.000	0.779	0.501
**Avg. var. extrac. (AVE)**	1.000	0.263	0.394
**Full collinearity. (VIFs)**	1.011	1.009	1.018
**Q** **-squared**	0.038		
